# Sterile innate immune mechanisms in neurodegenerative diseases

**DOI:** 10.1016/j.jbc.2025.111039

**Published:** 2025-12-11

**Authors:** Alyssa Ealy, Amanda M. Serapiglia, Nikhil Panicker

**Affiliations:** 1 Department of Neurosciences, Cleveland Clinic Research, Cleveland, Ohio, USA; 2 Department of Neurosciences, Case Western Reserve University School of Medicine, Cleveland, Ohio, USA; 3 Cleveland Clinic Lerner College of Medicine of Case Western Reserve University, Cleveland, OH, USA

**Keywords:** neurodegeneration, neuroinflammation, microglia, astrocytes, inflammasomes, cGAS/STING, lipid droplets

## Abstract

Neurodegenerative diseases are characterized by the dysfunction and death of susceptible neuronal populations. Increasing evidence has demonstrated that sustained neuroinflammation and activation of innate immune complexes underlie neurodegeneration, worsening disease progression and outcomes. Sterile inflammation (which occurs in the absence of infection) can be triggered by neurodegenerative disease–associated misfolded proteins. These studies highlight the need to decipher the complexities of innate immune signaling mechanisms and their contribution to neuropathology. In this review, we focus on major neurodegenerative diseases that have a well-documented neuroinflammatory component: Alzheimer’s disease, Parkinson’s disease, amyotrophic lateral sclerosis, multiple sclerosis, and frontotemporal dementia. In the context of these diseases, we discuss recent advances in innate-immune mechanisms that have been demonstrated to partake in disease progression and neurodegeneration. These include evolutionarily conserved innate-immune signaling complexes whose uncontrolled activation amplifies neurodegeneration, damaging lipid droplets that accumulate within myeloid cells and prevent their ability to clear toxic protein aggregates, as well as genome-wide studies–implicated genes/proteins. The individual and/or concerted actions of these pathways could be leveraged to rationally target the pathogenesis or progression of neurodegenerative diseases.

Neurodegenerative diseases are a diverse group of central nervous system disorders typified by the progressive and irreversible loss of neuron populations. Unable to be replenished like other cell types in the body, the death of these cells results in the breakdown of neuronal circuits, resulting in the onset of cognitive, behavioral, and/or motor deficits. Alzheimer's disease (AD) and frontotemporal dementia (FTD) are examples of degenerative brain disorders that involve loss of cognitive and executive brain function whereas Parkinson’s disease (PD), amyotrophic lateral sclerosis (ALS), and multiple sclerosis (MS) are major motor neurodegenerative diseases. Cumulatively, these disorders affect almost 2% of the global population, with disease incidence increasing year over year (1). Disease modifying therapies do not currently exist for the great majority of these disorders, and most existing drugs target symptoms rather than the underlying cellular processes that drive disease progression.

Despite the unique pattern of neuropathology, behavioral deficits, and neuron death in each of the diseases, there exists a great degree of overlap in the manifestation of these symptoms across their spectrum. For instance, > 80% of PD patients exhibit cognitive dysfunction/dementia 20 years post diagnosis (2). Identifying and understanding the conserved molecular underpinnings that underlay molecular dysregulation in neurodegenerative disorders could reveal new therapeutic targets to slow disease progression.

A unifying neuropathological feature among disparate neurodegenerative diseases is the presence of misfolded, aggregated proteins. Proteinaceous inclusions, such as amyloid-beta (Aβ)-rich senile plaques in AD and alpha-synuclein–rich Lewy bodies in PD, have long been reported upon postmortem observations in diseased tissues. Recent research has identified the presence of cross or mixed pathology across neurodegenerative disorders. For instance, tau tangles are present in ∼30% of the FTD cases but have also been observed in both AD and PD, leading to their classification as secondary tauopathies (3). Such overlapping neuropathology in diseases with unique pathological features and disparate affected brain regions is summarized in Figure 1*A*. It is currently unknown whether mixed proteopathy underlies or bears correlation with symptom overlap across different neurodegenerative diseases.

**Figure 1:**
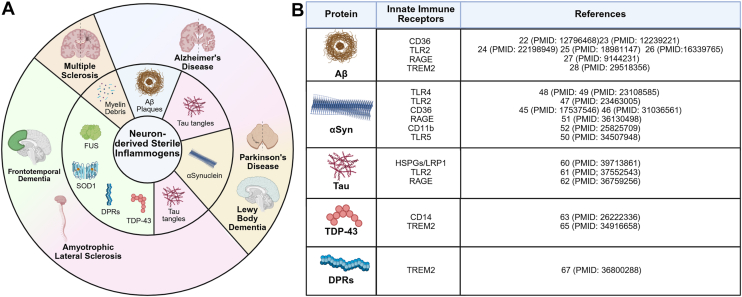
**Neurodegenerative disease–associated protein aggregates serve as sterile inflammogens**. While neurodegenerative disorders are disparate diseases with unique symptoms, common pathologic features are observed including chronic neuroinflammation and the presence of neuron-derived protein aggregates. *A*, sterile inflammation is ignited by nonpathogenic sources that occur in neurodegenerative disease where proteinaceous inclusions trigger activation of resident immune cells. Recent studies have demonstrated cross pathology between these diseases. AD brains show the presence of Aβ plaques and tau tangles. Tau tangles are not limited to AD but also appear in PD and ALS. PD patients also have αSyn-rich Lewy bodies which are also present in other brain regions in Lewy body dementia among other synucleinopathies. ALS and FTD share several pathogenic proteins such as TDP-43, DPRs, SOD1, and FUS which may present alone or present in combination with each other. Myelin debris serves as a sterile inflammogen in the central nervous system of MS patients. Together, these aggregated proteins activate several key innate immune mechanisms in glial cells which mount destructive immune responses that contribute to neurodegeneration. *B*, aggregated proteins present in neurodegenerative diseases activate several key receptors on microglia to trigger innate immune pathways. Each aggregated protein species interacts with a unique set of receptors. The downstream consequences of PRR activation by sterile inflammogens are explored in subsequent figures (24). AD, Alzheimer's disease; ALS, amyotrophic lateral sclerosis; Aβ, amyloid beta; FTD, frontotemporal dementia; FUS, fused in sarcoma RNA binding protein; MS, multiple sclerosis; PD, Parkinson’s disease; PRR, pattern recognition receptor; SOD1, superoxide dismutase 1.

Neuroinflammation, characterized by the aberrant activity/function of resident and/or infiltrating immune cells, is another conserved pathogenic feature of neurodegenerative disease that has come into focus as a potentially druggable disease modality. Evidence for neuroinflammation in neurodegenerative disorders was first identified in the 1980s, when reactive microglia and astrocytes were identified in AD and PD postmortem brains (4, 5, 6). Since these inceptive discoveries, more studies have confirmed increased levels of microglia (microgliosis) often in proximity to protein aggregates. Expanding upon the initial findings of chronic neuroinflammation in AD and PD, gliosis was confirmed in other neurodegenerative disorders including ALS (7), FTD (8), and MS (9, 10). Neurodegenerative disease–associated protein aggregates can mediate hyperactivation of microglial innate-immune complexes, triggering immunopathology-amplified toxic responses in the central nervous system (CNS) (Fig. 1*B*). Since these responses are not driven by pathogenic infection, they constitute examples of sterile neuroinflammation. Neurons express receptors for cytokines produced by microglia, resulting in the release of stress signals and initiation of apoptosis. Studies into the protective effects of nonsteroidal anti-inflammatory drugs and other anti-inflammatory therapeutics have shown some disease-modifying benefit. Moreover, MS therapeutic research has shown that drugs targeting inflammation are viable treatment options that can alter disease course. More recently, unbiased genome-wide association studies (GWAS) designed to identify risk factors for neurodegenerative disease onset in large cohorts of patient populations have identified unique neurodegenerative disease–associated risk factors that encode genes implicated in neuroimmune function (11, 12, 13). These studies, coupled with single-cell/single nuclei analysis from postmortem AD/PD brains, as well as studies utilizing rodent and induced pluripotent stem cell models have elucidated brain-resident innate immune signaling mechanisms that contribute to the neurodegenerative process.

Overall, despite the diversity of neurodegenerative disorders with regards to neuropathology and functional consequences including behavioral or cognitive dysfunction, several conserved CNS-specific neuroinflammatory signaling mechanisms contribute to disease pathogenesis. These include the cyclic GMP-AMP synthase (cGAS)/stimulator of interferon genes (STING) and inflammasome pathways, lipid droplets, and GWAS-implicated gene products that function in a conserved signaling pathway. The purpose of this review article is to highlight the progress that has been made in identifying novel innate immune pathways that have been implicated in neurodegenerative disorders.

## Inflammasome hyperactivation in neurodegenerative disease

**Figure 2:**
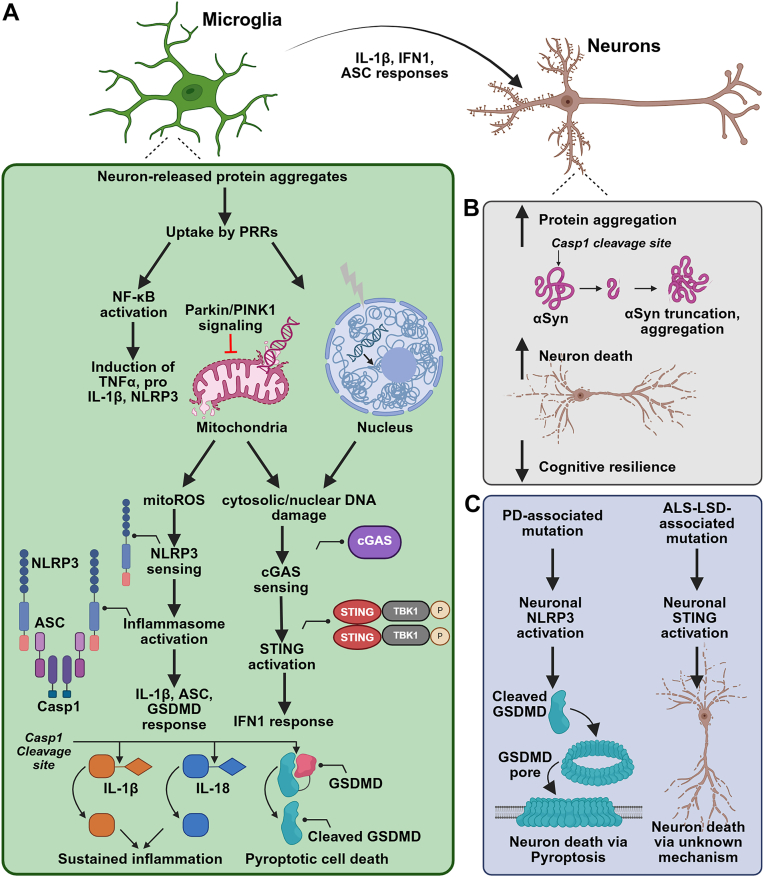
**STING and NLRP3 hyperactivation amplify neuropathology and neurodegeneration**. *A* and *B*, neurodegenerative disease–associated protein aggregates can be uptaken by microglial pattern recognition receptors (PRRs), leading to NF-κB responses, mitochondrial DNA/ROS release, and nuclear DNA escape. This leads the assembly of innate-immune assemblies such as the NLRP3 inflammasome and the cGAS/STING pathways, leading to IL-1β, GSDMD, and type-1 IFN responses that can directly result in diminished neuronal cognitive resilience, amplify protein aggregation, and neuron death. *C*, more recent work shows that PD/ALS-associated mutations result in neuron-resident NLRP3/STING responses, which can engage cell-autonomous cell-death mechanisms. ALS, amyotrophic lateral sclerosis; GSDMD, Gasdermin D; IFN, interferon; PD, Parkinson’s disease; STING, stimulator of interferon genes.

Inflammasomes are cytosolic multiprotein complexes that recognize pathogen- or damage-associated molecular patterns (PAMPs or DAMPs, respectively). These complexes consist of a PAMP/DAMP sensor or receptor protein, the adaptor protein ASC (Apoptosis-associated Speck Like Protein containing a Caspase-recruitment domain), and the effector protease Caspase-1 (Casp1). Inflammasome activation culminates in the Casp1-mediated processing and secretion of the cytokines IL-1β and IL-18 (14). Inceptive studies identified NLRP3 and NLRP1 as *bona fide* inflammasome receptors that facilitated Casp1 activation and IL-1β cleavage (15). Subsequent studies discovered other cytosolic inflammasome receptor proteins that could sense diverse ligands, expanding the innate-immune toolkit to respond to a plethora of stimuli (14). Despite being a prospective pathogen sensing/dispatching mechanism, mutations in the inflammasome receptor gene, *NLRP3*, has been shown to lead to autoimmune disorders (16, 17). To prevent unintended overactivation of the NLRP3 inflammasome, cells developed a failsafe mechanism in which two separate and independent signals are required to cleave Casp1. NLRP3 expression in many myeloid cells is insufficient to recruit ASC and can be induced by a "priming" signal that induces its transcriptional expression (18, 19). Next, a second "activating" signal triggers NLRP3-mediated ASC recruitment and Casp1 activation. Casp1 then cleaves pro-IL-1β and pro-IL-18 to their mature forms (Fig. 2*A*). Hyperactivation of the NLRP3 inflammasome has been reported in multiple peripheral immune diseases such as systemic lupus erythematosus, gout, and arthritis. The molecular components that trigger inflammasome priming and activation varies between diseases, with both sterile (uric acid crystals, nucleic acids, and mitochondrial reactive oxygen species (20)) and nonsterile (bacterial muramyl dipeptides and lipopolysaccharide) sources serving as potent NLRP3 activators. Emerging lines of evidence indicate that NLRP3 inflammasome hyperactivation can amplify neuropathology and neurodegeneration in diverse neurodegenerative disorders (14). In the sections below, we will summarize the mechanisms and consequences of this process.

### NLRP3 inflammasome activation in AD

Early studies identified IL-1β and IL-18 upregulation in postmortem AD brain tissue, well before their association as inflammasome-driven cytokines (21). AD-associated fibrillar Aβ was the first misfolded protein shown to act as an NLRP3-activating agent, eliciting IL-1β secretion and the formation of oligomerized ASC specks in lipopolysaccharide-primed primary mouse microglia through interactions with pattern recognition receptors (PRRs), CD36, TLR2, RAGE, and TREM2 (Fig. 1*B*) (22, 23, 24, 25, 26, 27, 28, 29). Subsequent *in vivo* studies reported elevated NLRP3 responses in postmortem cortical and hippocampal tissue from AD patients (30). Additionally, NLRP3 and Casp1-deficient mice were remarkably neuroprotected from amyloid-neuropathology, neurologic, and memory deficits when crossed with 5XFAD mice (which express five AD-linked mutations and progressively form Aβ plaques accompanied by gliosis) (30). These results were supported by studies utilizing the NLRP3 inflammasome-specific inhibitor MCC-950 (31) and the Casp1 inhibitor VX-765 (32) in mouse AD models. Taken together, these studies provide strong evidence for hyperactivation of the NLRP3 inflammasome in response to amyloid plaques both *in vitro* and *in vivo*.

IL-1β signaling modulates many cellular processes in a healthy brain including synapse maintenance, beneficial acute neuroinflammation, neuronal differentiation, and long-term potentiation (33). While beneficial at homeostatic levels, elevated IL-1β in the brain parenchyma following NLRP3 complex hyperactivation can have deleterious consequences. Conflicting data link IL-1β signaling with AD neuropathology. On the one hand, IL-1β can induce neuronal apoptosis *via* c-Fos upregulation (34, 35). Its relationship with AD-associated proteopathy is more complex—IL-1β can mitigate Aβ load by inducing glial cell proliferation and consequent phagocytosis, resulting in Aβ clearance (36, 37). Conversely, IL-1β–overexpressing mice exhibit enhanced tau phosphorylation due to activation of tau kinases, p38MAPK and GSK3β (36).

While IL-1β responses constitute the best characterized outcome of canonical NLRP3 inflammasome activation, recent studies have identified alternate inflammasome-driven mechanisms in AD. Inflammasome-activated microglia were found to upregulate and secrete the adaptor protein ASC, which associated with extracellular Aβ, cross-seeding, and hastening its aggregation (38). ASC and Aβ can form co-aggregates, potentiating each other's inflammasome-activating properties by broadening the scope of cell surface receptor recognition and downstream inflammatory responses (39). NLRP3 inflammasome assembly is also known to result in the proteolytic activation of the protein GasderminD (GSDMD), which then initiates a form of inflammatory cell death called pyroptosis by forming pores in the cell membrane (40). Microglial release of ASC specks was shown to ensue following GSDMD activation, suggesting that pyroptotic microglial death could contribute to AD-associated neuropathology (Fig. 2, *A* and *B*) (39). However, it is unclear whether the increased release of lactate dehydrogenase, which serves as a surrogate for assessing cell death in this system, is more reflective of transient membrane permeabilization rather than overt pyroptosis.

### NLRP3 inflammasome activation in PD

Early evidence for inflammasome activation was inferred in sporadic PD when polymorphisms in the IL-1β gene were associated with PD diagnosis (41). Subsequent studies found that IL-1β increases the susceptibility to dopaminergic neuron degeneration, driving disease progression (42). Lewy bodies, which are cytosolic neuronal inclusions rich in misfolded αSynuclein (αSyn), constitute the major PD neuropathological hallmark. A single intrastriatal injection of α Syn preformed fibrils (PFFs) initiates the templated misfolding of endogenous α Syn, culminating in progressive proteopathy, neurodegeneration, and the onset of PD motor deficits (43). As with fibrillar Aβ, αSyn PFFs were found to induce microglial NLRP3 inflammasome assembly and consequent inflammasome-marshalled neurodegenerative responses (44, 45). Several membrane-bound receptors were found to drive αSyn-marshaled microglial responses, either by direct engagement or through indirect/transient interactions that mediated downstream NFκB activation (Fig. 2*A*). These include the surface receptor CD36 (46, 47), TLR2 (48), TLR4 (49, 50), TLR5 (51), RAGE (52), and CD11b (53) (Fig. 1*B*). Subsequent studies confirmed αSyn PFFs induced NLRP3 induction, assembly, and IL-1β release in lipopolysaccharide-primed primary human microglia (54). NLRP3 activation in PD is not limited to myeloid cells. Mutations in the *PRKN* gene, which encodes for the Parkin E3 ubiquitin ligase, cause familial (autosomal recessive) PD. Loss of Parkin function results in neuronal NLRP3 accumulation and subsequent neurodegeneration (Fig. 2*C*) (55). The mechanisms that link microglial inflammasome activation with neuronal proteopathy in PD models remain largely undercharacterized.

### NLRP3 inflammasome activation in ALS-FTD

Because of overlapping genetics and neuropathological features, ALS and FTD are believed to exist on a continuum. Multiple pathogenic aggregated proteins, including Tau, superoxide dismutase 1 (SOD1), fused in sarcoma RNA binding protein, and TAR DNA–binding protein 43 (TDP-43), are shared between these disorders. The spatiotemporal manifestation of symptoms and neuropathology dictates ALS/FTD diagnosis—ALS results in the death of neurons in the spinal cord, brainstem, and motor cortex, and FTD entails neuronal degeneration within the temporal lobes, insular cortex, and the cingulate cortex (56, 57).

Mice expressing humanized P301S Tau were shown to upregulate IL-1β (58). IL-1β was found to exacerbate tau pathology in AD models, further suggesting that inflammasome signaling may contribute to the pathophysiology of tauopathies including FTD (59). Recombinant human truncated Tau seeds as well as FTD brain-derived Tau aggregates were demonstrated to be potent NLRP3 inflammasome activators in cultured microglia and in transgenic mutant-Tau overexpressing mice (60, 61). Tau is recognized by several PRRs on microglia including HSPGs/LRP1 (62), TLR2 (63), and RAGE (64) (Fig. 1*B*). Global as well as microglia-specific ablation of NLRP3 inflammasome components blunted Tau aggregation in FTD models (59, 61). Tau hyperphosphorylation precedes and drives its aggregation, and inflammasome assembly was demonstrated to contribute to this process by amplifying tau kinase and phosphatase activity (59). Evaluation of NLRP3 inflammasome activation in ASC-deficient Tau-overexpressing mice showed diminished ASC specks, Caspase-1 cleavage, and IL-1β (59).

As with other neurodegenerative disease–associated proteins, aggregates of TDP-43 were found to act as an efficient activating signal to license NLRP3-induced IL-1β release in microglia (65, 66). TDP-43 engagement by the surface receptor CD14 facilitates NFkB signaling, enabling NLRP3 complex priming (Fig. 1*B*) (65). On the other hand, TDP43 can also interact with TREM2, increasing TDP43 phagocytosis and improving neuronal deficits (67). In mutant TDP43 mice, NLRP3, Caspase-1, and ASC expression were elevated in spinal cord microglia (68). Dipeptide repeats (DPRs) have been associated with ALS and have been found to elicit a neuroinflammatory response in ALS through TREM2-mediated internalization (69). Transfection of PR_50_, a DPR species, into human microglia elicited NLRP3 inflammasome activation, indicating that DPRs can prime and activate the inflammasome (70). Conditioned media from PR_50_-exposed microglia induced neuronal cell death, which was attenuated upon pharmacological NLRP3 inhibition. Another species of DPRs, GA_50_, has also been shown to prime and activate the NLRP3 inflammasome upon transfection into microglia (71). A mouse model of ALS entailing AAV-GR_100_ overexpression exhibited molecular signatures of microglial NLRP3 complex hyperactivation (72). NLRP3-deficient mice were protected from neuropathologic and neurodegenerative deficits in this paradigm (72). In postmortem ALS tissue, an upregulation of microglial cleaved GSDMD in the white matter and in the motor cortex, suggesting increased NLRP3 activity in disease (73). The SOD1^G93A^ ALS model recapitulates ALS proteopathy. Spinal cord astrocytes, rather than microglia were initially identified as the primary inflammasome-competent cells in SOD1 mutant ice (74), but conflicting studies suggest that ALS-associated SOD1 aggregates are potent inflammasome activators (68).

### NLRP3 inflammasome activation in MS

Multiple sclerosis is characterized by autoimmune demyelination and subsequent neurodegeneration in the brain and spinal cord. Demyelination ensues following myelin-reactive CD4+ T cell infiltration into the brain. In addition to this pathologic adaptive immune response, reactive microglia have been found near MS lesions, amplifying local innate and adaptive neuroinflammatory responses. MS can be modeled in mice by administering cuprizone, which effectuates demyelination, or by immunizing mice with myelin peptides, resulting in experimental autoimmune encephalomyelitis (EAE).

The cuprizone model of demyelination has shown activation of microglia that is accompanied by upregulation of NLRP3, IL-18, and IL-1β (75, 76). NLRP3-deficient mice exhibited reduced microglial infiltration into the corpus callosum, accompanied by attenuated demyelination (75).

In the EAE model, elevated transcripts of NLRP3 were observed before the onset of disease (77, 78). The pharmacological NLRP3 inhibitor MCC950 alleviated reactive microgliosis, neuron loss, and demyelination in the EAE mouse model of MS (79, 80, 81). Studies utilizing inflammasome-deficient transgenic mice phenocopied these results, demonstrating blunted EAE-induced disease severity, neuron loss, and peripheral cell infiltration into the CNS (78, 82, 83). RNA-seq analysis from patients with primary progressive multiple sclerosis showed upregulation of NLRP3 and IL-1β (81). Subsequently, the NLRP3 complex assembly was found to require NLRC4 for activation and assembly in bone marrow–derived macrophages and glial cells in the cuprizone mouse model of progressive demyelination, suggesting that multireceptor inflammasomes could function in unison to mount consequent Casp1 responses (84). Subsequent research suggested that NLRP3-mediated IL-1β and IL-18 in EAE mice amplify disease pathology by facilitating T-cell infiltration chemotaxis (85).

### AIM2 inflammasome in neurodegenerative disease

Absent in melanoma 2 (AIM2) is an inflammasome receptor that detects dsDNA within the cytosol, resulting in consequent Casp1 and IL-1β responses. Thus far, limited evidence depicts AIM2 roles within the central nervous system. Neuron-specific AIM2 was found to orchestrate homeostatic, pyroptotic neuronal dieback during development (86). Microglia-specific AIM2 inflammasome hyperactivation was demonstrated in the APP/PS1 AD model (87) and found to contribute to synaptic and cognitive impairments in an Aβ injection AD model (88). Conversely, anti-inflammatory actions of AIM2 were documented in T cells as well as myeloid cells in the EAE model of MS, acting to downregulate AKT activation and consequent pro-inflammatory cytokine induction (89). AIM2 inflammasome activation (which did not culminate in a detectable IL-1β response) was observed in astrocytes, but the consequences of this remain largely unknown (90).

Overall, there is abundant evidence that proteinaceous aggregates present in neurodegenerative diseases can trigger hyperactivation of inflammasomes in glial cells. The consequences of NLRP3 overactivation are deleterious, leading to accumulation of protein aggregates, elevated levels of cytokines that propagate inflammation, and induction of cell death pathways. The consequences of AIM2 activation can provide a protective effect that goes beyond canonical ASC speck formation and IL-1β secretion, highlighting diversity in inflammasome functions in neurodegenerative diseases. While the products of canonical NLRP3 inflammasome activation are well documented, several unanswered questions remain, including how microglial cells can mount inflammasome responses without the concomitant engagement of pyroptotic death. Additionally, inflammasome sensors may work synergistically to modulate neuroinflammation, which could lead to novel findings in neurodegenerative diseases. Lastly, therapeutics that function as inflammasome antagonists have been demonstrated to attenuate proteopathy and neurodegeneration in multiple neurodegenerative disease models, but it remains to be seen whether these findings will translate to human studies.

## cGAS/STING signaling in neurodegenerative disease

Since DNA is customarily sequestered in membrane-bound organelles, its presence in the cytosol indicates prospective infection by pathogens. This mobilizes the assembly of innate-immune, PAMP-sensing platforms. Briefly, cytosolic DNA is recognized by the protein cyclic GMP-AMP synthase (cGAS), stimulating production of 2′3′-cyclic GMP-AMP (2′3′-cGAMP). Elevated levels of 2′3′-cGAMP activate STING, a protein primarily localized to the ER (91). This process culminates in the induction of type-1 interferons (IFN-1) (91, 92, 93). cGAS senses dsDNA in a length-dependent manner by binding to the DNA phosphate backbone (94). Once activated, it catalyzes the formation of 2′3′-cGAMP, a cyclic dinucleotide (95, 96). 2′3′-cGAMP then acts as a secondary messenger to activate STING (Fig. 2*A*) (96). Activated STING undergoes a conformational change and translocates to the perinuclear region of the ER as well as the Golgi (97, 98). The C terminus of STING recruits tank-binding kinase 1 (TBK1), promoting TBK1 autophosphorylation, which in turn serine-phosphorylates STING. STING phosphorylation leads to the recruitment and phosphorylation of interferon response factor 3 (IRF3) by TBK1, prompting nuclear translocation of pIRF3 and transcriptional induction of IRF3 targets, including IFNβ, IL-6, and IL-12 (97, 99, 100, 101, 102). Additionally, STING can drive the activation of NF κ B to upregulate pro-inflammatory cytokine transcription (93, 101, 103, 104).

The cGAS/STING complex plays a critical role in peripheral innate immune regulation by sensing cytosolic DNA and inducing the production of IFNβ to mount antiviral responses (91, 98, 102, 105, 106). cGAS-generated cGAMP can be transferred from one cell to neighboring cells through gap junctions, serving to propagate cGAS/STING activation in recipient cells, elucidating a mechanism enabling the spread of antiviral immunity to adjacent cells (96).

More recently, cGAS/STING hyperactivation was shown to partake in age-associated pathology, driving chronic inflammation in response to the accumulation of mitochondrial DNA in the aging brain (107). The hyperactivation of this pathway, which ostensibly evolved to effectuate pathogen clearance, has recently been shown to amplify and partake in AD, PD, and FTD-associated neurodegeneration pathologies. Herein, we aim to elucidate cGAS/STING activation mechanisms and consequences in the context of neurodegeneration.

### cGAS/STING in AD

Analysis of cerebral and spinal cord tissue in postmortem AD human samples and 5xFAD mice revealed molecular signatures of cGAS/STING pathway hyperassembly, which led to the buildup of Aβ pathology and neurodegeneration (108, 109). Similarly, increased cGAS/STING assembly was observed in a mouse tauopathy model, and the activation of this pathway was amplified in the presence of AD-associated mutations (110). The consequences of cGAS/STING activation in AD are far-reaching, resulting in decreased Aβ phagocytosis, pro-inflammatory cytokine release, and attenuated pro-survival neuronal transcriptional signaling. cGAS-deficient 5xFAD mice exhibit improved cognitive function and decreased Aβ burden, suggesting that this pathway could be leveraged for therapeutic targeting (108, 111). A small molecule STING inhibitor, H-151, was found to block IFN responses, alleviating pathologic features and memory deficits in AD mouse models (108, 112). Furthermore, pathogenic Tau has been shown to activate cGAS and consequent IFN-1 responses in microglia. Pharmacological inhibition of cGAS in P301S mice restored memory deficits, protected against synapse loss, and enhanced the neuronal transcriptional network of myocyte enhancer factor 2c, a gene that has been implicated in cognitive resilience (Fig. 2*B*) (113, 114). Intriguingly, the rare Christchurch mutation, APOE R154S, has been shown to have protective effects against AD *via* cGAS/STING-IFN inhibition in microglia. This supports the therapeutic potential of targeting the cGAS/STING–IFN-1 axis to improve pathological and cognitive deficits that are prevalent in AD patients (Fig. 2*B*) (115). Recent work has also demonstrated that polyglutamine binding protein 1 senses extracellular 3R/4R Tau proteins, thereby triggering cGAS and consequent NFκB responses (116). However, the mechanisms by which STING-activated microglia contribute to neuron death in AD models is still an active area of investigation, with some studies suggesting that this occurs *via* microglia-mediated induction of alternate-fate–directed neurotoxic astrocytes (108), increasing neuron vulnerability following IFN-mediated downregulation of cognitive resilience transcription networks (Fig. 2*B*) (113). It remains unknown whether microglial cGAS/STING assembly and/or consequent IFN responses directly engage cell death pathways within neurons.

### cGAS/STING in PD

As with other neurodegenerative disease–associated protein aggregates, α Syn fibrils can elicit cGAS/STING assembly.α Syn-PFF–injected mice upregulated γH2AX (an indicator of nuclear dsDNA damage), cGAS, STING, and pTBK1 as well as STING-dependent IFNβ levels (117). Mice lacking STING were protected from α Syn PFF-induced dopamine neuron loss (117). Mitochondrial dysfunction is prominent in PD and other neurodegenerative disorders (118). The familial PD-linked proteins Parkin and PINK1 function in a conserved signaling pathway that mediates mitochondrial quality control (119); Parkin and PINK1 have been shown to mitigate mitochondrial stress *in vivo* by preventing the generation of circulating mtDNA *via* mitophagy (120). mtDNA stress ensuing from Parkin/PINK1 deletion results in STING activation and NFκB signaling (Fig. 2*A*) (120). Elucidating the signaling mechanisms that trigger the cytosolic escape of nuclear and/or mtDNA, which act as cGAS ligands, is an active area of research. Addressing how assembly of the cGAS/STING pathway contributes to PD-associated dopaminergic neuron death and/or neuropathology is a relatively underexplored research avenue though. Intriguing models utilizing a constitutively active STING variant suggested that STING activation results in NLRP3 inflammasome assembly and facilitates endogenous α Syn aggregation and dopaminergic neurodegeneration *in vivo* (121). These results suggest that STING assembly is sufficient to drive PD-associated proteopathy. Inhibition of inflammasome activation but not type-1 IFN signaling additionally blunted striatal oxidative stress and nitrite production in this model (121). Thus, STING/NLRP3 interdigitation may play a role in the progression of neuropathology and dopaminergic neurodegeneration in PD, although the mechanisms that facilitate this remain undercharacterized.

### cGAS/STING in MS

STING-deficient mice did not exhibit amplified EAE scores post immunization and exhibited attenuated scores during early disease course (122). Congruent with the neuroprotective roles of STING signaling in MS, microparticle-encapsulated cGAMP could mediate neuroprotection in EAE in a STING-dependent manner, specifically decreasing disease score and reduced relapses (123). cGAMP microparticles induced the production of anti-inflammatory cytokines, IL-10 and IL-27, and attenuated cellular infiltration into the spinal cord (123). The cGAMP transporter protein LRRC8C facilitated STING activation within T-cells, leading to an attenuation of T-cell responses. LRRC8C-deficient mice showed reduced STING activation and amplified disease scores following EAE induction (124). Type-1 IFNs were the inceptive class of therapeutics used as MS therapeutics (125). In line with this, an FDA-approved antiviral drug, Ganciclovir was found to orchestrate neuroprotective STING-marshalled IFNβ responses in EAE, suggesting that tonic STING signaling may limit autoimmune demyelination. Taken together, these results break with conventional observations made in the models of AD and PD, delineating neuroprotective roles for cGAS/STING activation in MS. However, more recent work has demonstrated that unconventional STING signaling within neurons led to neuronal vulnerability to excitotoxic stress in EAE (126). A discussion of this work will continue in the next section. Cumulatively, these data paint a complex, sometimes conflicting picture of STING functions in MS—on the one hand, its activation in autoimmune neuroimmune settings can marshal neuroinflammation-dampening signaling, while its noncanonical roles within neurons can potentiate MS-associated neurodegeneration. More detailed, cell type–specific exploration of STING-marshalled signaling may provide insight and lead to a resolution of these apparently contradictory findings.

### Cell-autonomous cGAS/STING in neurodegenerative disease

While assembly of the cGAS/STING pathway in myeloid cells of the brain has been considered a canonical response to pathogenic DAMPs/PAMPs, neuron-specific roles of STING in the models of neurodegenerative disorders have been noted as well. Noncanonical STING activity can initiate cGAS-independent ferroptotic cell death in neurons *via* store-operated calcium entry in response to IFNγ or glutamate (126). As mentioned previously, small molecule–mediated cGAS/STING inhibition blunted clinical deficits in the EAE model. Conversely, neuron-specific STING deletion improved EAE disease course and neurodegeneration, demonstrating that noncanonical, cell-autonomous STING may play an important role in MS (126). Canonical cGAS/STING activation was observed in ALS/FTD patient iPSC-derived neurons, layer V cortical motor neurons in *C9orf72* repeat expansion transgenic mice, as well as cortical and spinal motor neurons in postmortem ALS tissue, demonstrating that neuronal STING activation, resulting in *Tnfα*, *Il6*, and *Cxcl10* production, drives pathogenesis in ALS/FTD models (127). More recent work has identified lysosomal storage disorder–associated mutations effectuate cell-autonomous cGAS/STING assembly in neurons, leading to their death (Fig. 2*C*) (128).

Intriguingly, *TBK1* (recruited by STING in the response to PAMPs) has recently been identified as a genetic risk factor for both ALS and FTD. Loss-of-function mutations in *TBK1* have been documented in C9orf72, TDP-43, and SOD1 ALS/FTD cases (129, 130). It is unclear whether neuropathology and neurodegeneration that ensue under these circumstances entail a reduction in canonical or noncanonical STING signaling. Accumulation of TDP-43 within neurons is a feature of ALS and was found to engage canonical cGAS/STING assembly within diseased motor neurons, acting as critical mediators of TDP-43–mediated deficits (131). It is unclear whether the ability of neurons to mount canonical and noncanonical STING-initiated signaling is representative of an evolutionarily conserved innate-immune remnant or whether STING component proteins serve physiologic neuronal functions that are subverted in the settings of neurodegeneration.

## Lipid droplets

**Figure 3:**
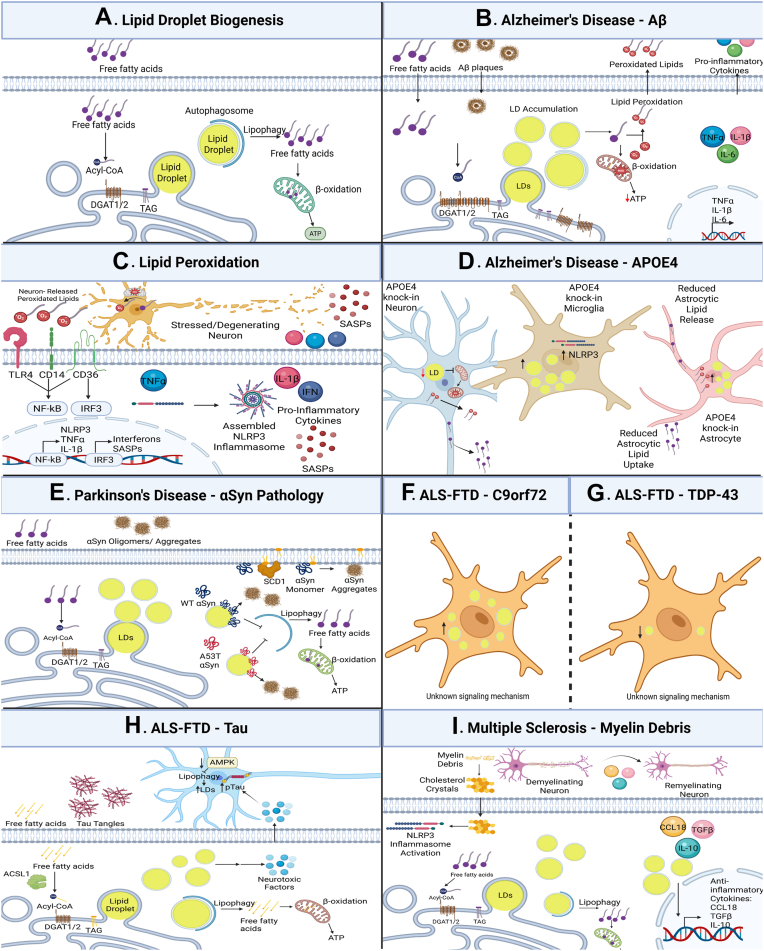
**Lipid droplet dysregulation in neurodegenerative diseases**. *A*, free fatty acids are imported into the cell. DGAT1 and DGAT2, transmembrane ER proteins, catalyze the conversion of diacylglycerol and fatty acyl CoA to neutral triglycerides, which collect in the ER membrane before it buds off to form lipid droplets. Autophagosomes encapsulate lipid droplets to break down triglycerides and cholesterol esters to free fatty acids that are utilized by mitochondria to produce ATP. *B*, microglia accumulate lipid droplets in AD with Aβ pathology. Mitochondrial dysfunction increases ROS leading to increased lipid peroxidation, driving release of pro-inflammatory cytokines TNFα, IL-1β, and IL-6. *C*, neuronal stress results in neuronal lipid peroxidation and release. Released peroxidated lipids can interact with several microglial receptors including TLR4, CD14, and CD36 to activate pro-inflammatory transcription factors culminating in NLRP3 inflammasome TNFα and interferon responses. Recruitment of IRF3 to the nucleus also induces transcription and subsequent expression of senescence-associated secretory phenotype proteins. *D*, in AD driven by the APOE4 genotype, reduced neuronal lipid droplets attenuate lipid and peroxidated lipid release. Astrocytes and microglia accumulate lipids, leading to increased lipid peroxidation, NLRP3 inflammasome activation, and decreased astrocytic lipid release. Together, impaired neuron-glia lipid transport is observed with APOE4 expression. *E*, in αSynucleinopathies, LDs accumulate in microglia. Additionally, WT and A53T αSyn are inserted into the monolayer membrane surrounding LDs, impairing lipophagy and amplifying αSyn aggregation. SCD1 adds a double bond to fatty acids, loosening the membrane pack. This increases the time αSyn stays in the membrane and increases aggregation. *F*, in ALS-FTD with C9orf72, microglial LDs accumulate while (*G*) microglial LD levels are depleted in the settings of TDP-43 ALS-FTD. *H*, Tau pathology in ALS-FTD results in microglial LD accumulation and release of neurotoxic factors that increase tau phosphorylation. Tau pathology in neurons also reduces levels of AMPK, which inhibits lipophagy under normal physiological conditions, leading to increased neuronal LDs. *I*, cholesterol crystals from myelin debris result in LD generation, activating the NLRP3 inflammasome in microglia. Increased microglial LDs can increase anti-inflammatory cytokines CCL18, TGFβ, and IL-10. Together, neurodegenerative diseases demonstrate altered lipid homeostasis. AD, Alzheimer's disease; ALS, amyotrophic lateral sclerosis; AMPK, adenosine monophosphate-activated kinase; Aβ, amyloid beta; ER, endoplasmic reticulum; FTD, frontotemporal dementia; LD, lipid droplet; SCD, stearoyl-coA-desaturase.

Lipid droplets (LDs), originally identified in the 1890s (132), are ubiquitous, phospholipid monolayer-bound organelles that serve critical roles in brain homeostasis and disease. LDs ostensibly evolved to serve as an energy source in the absence or dearth of glucose (133), but more recent work has elucidated their roles in the settings of homeostasis and disease. LDs can alleviate lipotoxic and oxidative stress (134, 135). In contrast, dysregulated LD accumulation is harmful and can amplify pathology under various conditions (136). LD biogenesis initiates at the endoplasmic reticulum (ER), where the diacylglycerol acyltransferase enzymes DGAT1 and DGAT2 convert free fatty acids to triglycerides (137). After budding off the ER membrane, LDs become mature, phospholipid monolayer-enrobed LDs (Fig. 3*A*). LDs store energy in the form of neutral triglycerides and cholesterols, which can be broken down into free fatty acids. Free fatty acids can then be used by mitochondria for ATP generation under conditions of nutrient starvation (138, 139). Within the brain, these functions are intimately involved in the establishment of neuron-astrocyte metabolic coupling (140).

In his seminal treatise on the eponymous brain disorder, Alois Alzheimer considered the presence of “glial fatty saccules” within degenerating brain tissue a cardinal feature of AD neuropathology, along with neurofibrillary tangles and amyloid plaques (141). Despite these early observations, the roles that LDs play in neurodegenerative disease pathogenesis are only beginning to be elucidated and may mirror prior research in the field of cardiovascular disease/atherosclerosis wherein lipid accumulation within macrophages was shown to contribute to the process of atherogenesis in cardiovascular disease (142). In the settings of atherosclerosis, LD-laden foam cells exacerbated local inflammation and promoted plaque instability. Ablation of LD-laden senescent macrophages ameliorated disease course (143).

In a *Drosophila* model of photoreceptor neurodegeneration, LD accumulation within epithelial glia adjacent to neuron terminals preceded and contributed to neuron death, suggesting that LD accumulation could have deleterious consequences within the central nervous system (144). Subsequently, a meta-analysis of AD-associated risk factors identified through GWAS implicated lipid metabolism as an AD-associated genetic risk factor (145). Microglia in aged mice were found to accrue LDs. These LD-laden microglia upregulated transcriptional signatures associated with mitochondrial dysfunction, producing nitrites and reactive oxygen species (ROS), and were found to exhibit defects in phagocytic activity, coupled with amplified inflammatory responses (146). An unbiased CRISPR–Cas9 screen to identify drivers of LD generation within microglia identified multiple genes associated with neurodegenerative disease, including *GRN* (mutations which are associated with FTD) and *VPS35* (mutations associated with PD) (146). These findings set in motion the hypothesis that glial LD accumulation could drive neuropathology in brain disorders.

### LDs in AD

Postmortem AD brain hippocampal sections were found to exhibit a marked increase in overall LD density as well as microglia-specific LD abundance when compared with age-matched control samples. These results were phenocopied in the 5xFAD familial AD model, which exhibited a time- and sex-dependent (more prevalent in female mice) increase in microglial LD levels within the hippocampus and subiculum (147). Intriguingly, LD accumulation was found to precede neurodegenerative deficits.

Although these results, as well the findings described in the previous section, have rekindled interest in exploring how LDs drive disease pathology in AD, lipid dysregulation has long been implicated in AD pathogenesis and the most common form of the disease, late-onset AD (which occurs in individuals over the age of 65 (148)). Saturated lipids contained within lipoparticles secreted by alternate fate-directed astrocytes could directly mediate neuron death (149), although it remains to be verified whether this mechanism contributes to neurodegeneration in the mouse models of neurodegenerative disease. Transcriptomic profiling of LD-rich microglia revealed a significant increase in senescence-associated secretory phenotype–related proteins; a phenomenon in which senescent microglia release cytokines, chemokines, proteases, and growth factors, propagating inflammation and neurodegeneration 152 (152). Functionally, LD-laden microglia have altered transcriptomes that skew towards a pro-inflammatory and senescent state and exhibit reduced capacity to clear Aβ aggregates (146, 150, 151).

Following the observations of increased LD-laden glia in AD brains and in models of AD and taking into consideration how lipid dysfunction can amplify neuroimmune responses, it is conceivable that LD accumulation can be targeted as a potential therapeutic strategy. However, complete attenuation of LD biogenesis can also be toxic, demonstrating that LDs are necessary for cell homeostasis (144, 153). Using inhibitors of DGAT2 to diminish but not eliminate LD production in 5xFAD mice reduced Aβ plaque burden (Fig. 3*B*) (147).

As a method to combat increased oxidative stress generated by mitochondrial dysfunction, reactive oxygen species are adducted to free fatty acids generating peroxidated lipid species (Fig. 3*C*) (154). Neurons secrete free fatty acids as well as peroxidated lipids that can be phagocytosed by glia, contributing to glial-LD accumulation (144). Peroxidated lipids can act as ligands for PRRs, triggering and/or amplifying assembly of innate-immune pathways such as the NLRP3 inflammasome (155). Finally, a combination of the reasons could act synergistically to facilitate LD accumulation and consequent deficits.

Allelic variants of the apolipoprotein E (*APOE*) gene, namely *APOE2*, *APOE3*, and *APOE4*, are intimately associated with AD etiology, with *APOE4* genotype constituting the highest risk factor for late-onset AD (156, 157). The APOE protein has been characterized as a lipid transporter that mediates the binding of lipoproteins to cell surface receptors (158). APOE has also been found to exhibit dynamic, stress-inducible expression within CNS cell types (159). Following the discovery that glia-derived LDs could perpetuate neuron dysfunction and death and given the intimate association of APOE in regulating lipid metabolism, attention was turned to assessing whether these pathways were connected. APOE-deficient cells were found to exhibit diminished LD accumulation in response to cellular stressors, and APOE4 overexpressing *Drosophila* showed exacerbated photoreceptor neuron loss (160). Utilizing a humanized *APOE3/APOE4* knock-in mouse line, it was shown that neuronal expression of the *APOE4* allele attenuates LD generation, leading to diminished mitochondrial function (161). Astrocytic expression of the *APOE4* allele rendered the cells inefficient at clearing neuron-derived LDs, culminating in elevated lipid load and loss of their homeostatic functions (Fig. 3*D*) (161). LDs within *APOE4* knock-in astrocytes were more prone to peroxidation (162). More recently, a link was established between the *APOE* genotype and LD accumulation within AD brain tissue; Oil-red O neutral lipid staining revealed an APOE genotype–nested expression pattern of lipid bodies within postmortem brain tissue, with *APOE3/3* AD patients showing a significant induction in lipid bodies distributed within the brain parenchyma compared to age-matched control subjects, and *APOE4/4* AD patients showing a statistically higher expression over the *APOE3/3* subjects. The number of lipid bodies was negatively correlated with cognitive performance and positively correlated with Braak-staged neuropathology (151). Collectively, these studies provide considerable evidence of LD induction in the settings of AD, as well as evidence that LD induction correlates with AD neuropathological and cognitive deficits.

This pathology-associated increase in LDs could ensue for several reasons. One, increased endogenous LD synthesis *via* disease-associated induction of LD biosynthesis proteins. Congruent with this posit, DGAT2, which catalyzes the rate-determining step in triglyceride biosynthesis, was induced within microglia in 5xFAD mice and in postmortem AD brains (147). LDs may also accumulate due to a compromise in LD breakdown pathways; LDs are degraded by a form of selective autophagy termed lipophagy (163). In a rodent model of cognitive impairment, deficits in microglial lipophagy were found to drive LD accumulation (164).

Overall, considerable evidence suggests that AD-associated LDs contribute to neurodegeneration in AD, and strategies aimed at maintaining LD homeostasis may be a prudent approach to target lipid-induced neuroinflammation and subsequent neurodegeneration in AD.

### LDs in PD

The protein αSyn, best known as the primary component of Lewy bodies in PD in its aggregated form, was first characterized as a presynaptic protein with the tendency to exist within membranous niches (165, 166). The N-terminal domain of αSyn can bind lipids, anchoring it to the outer membrane of LDs (167). Interestingly, familial PD-causing mutant αSyn variants exhibit differential properties in their ability to integrate into LDs, with WT and A53T αSyn showing colocalization with LDs, while A30P αSyn remaining constrained within the cytoplasm. Furthermore, WT but not A53T αSyn inhibits lipolysis of LDs, suggesting that altered LD dynamics in the settings of PD could result following the loss of physiologic αSyn function (Fig. 3*E*) (167). Lipidomic profiling revealed that αSyn overexpression induced oleic acid, diglycerides, and triglycerides (168). Oleic acid was found to amplify αSyn aggregation and toxicity *via* induction of the protein stearoyl-CoA-desaturase, which adds double bonds to saturated fatty acids in lipid membranes (169), serving to increase αSyn membrane association and consequent fibrilization (168). Further work utilizing a *Drosophila* model suggested that αSyn interactions with LDs rendered it more resistant to proteinase-K digestion, indicative of aberrant, aggregation-prone conformation (170).

Glucocerebrosidase, encoded by the glucosylceramidase beta 1 (GBA) gene, is the highest genetic risk factor for developing PD and dementia with Lewy bodies, increasing the odds ratio of developing these diseases 5-10-fold. Localized to lysosomes, GBA catalyzes the breakdown of complex glycolipids to glucose and lipid products. Loss-of-function mutations in GBA result in the accumulation of complex and neutral lipids in the substantia nigra as well as increased levels of aggregated αSyn. Over 300 mutations in GBA have been identified, affecting lysosomal trafficking, enzymatic activity, interactions with αSyn, and lipid homeostasis (171). The most common GBA mutation associated with PD is the E326K variant, which does not alter lysosomal trafficking or glucocerebrosidase activity (as seen with other disease-associated mutations) but alters interactions with αSyn and lipids. E326K mutant iPSC neurons had increased levels of glucosylated cholesterols that increased their levels in LDs and promoted αSyn aggregation through membrane fluidity (172). The transgenic 3K αSyn mouse model expresses three *SNCA* point mutations (E35K, E46K, and E61K) that result in enhanced αSyn aggregation. This mouse model exhibits high LD and pS129 αSyn load that are diminished with GBA overexpression (173). It is likely that dysfunctional GBA reduces lipophagy through reduced lysosomal activity, leading to LD accumulation in PD. Assessment of LD abundance *via* neutral lipid staining of postmortem PD and age-matched brain tissue revealed cell type–specific alterations—while total neutral lipid content remained unchanged, dopaminergic neurons and microglia were found to exhibit significantly higher LD levels in PD ventral midbrain tissues. These results were recapitulated upon inhibition of GBA activity in mice (174).

Glycoprotein non-metastatic melanoma protein B (GPNMB) is a glycoprotein that plays a role in lipid metabolism. Initially discovered in cancer, GPNMB function has been further demonstrated in the induction of free fatty acid synthesis (175). Additionally, GPNMB has been shown to play a role in inflammation through induction of NFκB signaling in myeloid cells, including microglia (176). GPNMB was subsequently identified as a risk factor for PD (175). GPNMB-deficient iPSC neurons exhibited reduced internalization and phosphorylation of αSyn. While several studies have indicated a role for GPNMB in neuroinflammation, it is unclear if it is involved in pro- or anti-inflammatory signaling. A majority of studies have reported an anti-inflammatory role for GPNMB, demonstrating decreased secretion of microglial pro-inflammatory cytokines with GPNMB overexpression (177). However, knockdown of GPNMB in BV2 microglia resulted in decreased secretion of iNOS, TNFα, and IL-1β. Because of the connection between GPNMB in neuroinflammation and lipid synthesis in the periphery, future research could elucidate how GPNMB drives LD-mediated microglial activation.

In sum, these results demonstrate a reciprocally bidirectional relationship between PD-associated neuropathology and LD accumulation. Given the well-characterized biochemical roles of PD-associated proteins such as GBA1 and GPNMB in regulating lipid metabolism and/or dynamics and considering the commonalities and overlap in AD and PD pathophysiology, including and not limited to excess oxidative stress and sterile inflammation, it is intriguing to consider whether preventing LD accumulation in PD models and/or patients can be utilized to halt disease progression. As a final LD-associated link between AD and PD, the *APOE4* genotype was found to exacerbate αSyn pathology independently of beta amyloid or Tau (178, 179). We hypothesize that aberrant LD accumulation and LD-amplified αSyn aggregation in these settings could lead to this phenotype.

### LDs in ALS and FTD

Lipidomic analyses from postmortem tissues across the ALS-FTD continuum indicate LD dysregulation when compared to age-matched tissues, while showing inconsistent trends with regards to changes in different lipid types. For example, cholesterol esters, a main component of LDs, were reported to increase in ALS with no change observed in triglycerides (180). However, conflicting reports show elevated triglyceride levels in ALS patients (181, 182). These inconsistencies between lipid species may reflect changes affected by the continuum of disease pathology in ALS/FTD. Loss of C9orf72 was found to elicit LD accumulation due to disruption of lipophagy pathways (Fig. 3*F*) (183). Conversely, TDP-43 depletion resulted in the reduction of LDs (Fig. 3*G*) (184). The consequences of high LD load in microglia have also been shown to affect disease pathogenesis in ALS and FTD. For instance, LD-harboring microglia inefficiently clear aggregated Tau (185). These microglia can also induce pathology in surrounding neurons. Conditioned media from LD-harboring microglia induced tau phosphorylation when added to neurons, suggesting that LD-accumulating microglia release neurotoxic factors that induce a disease state (151). However, it remains unknown if the released neurotoxic factors consist of cytokines, lipids, or other pathology-inducing factors. In an early onset ALS model, a reduced LD load was observed due to lysosomal dysfunction (186). The P301S Tau transgenic mouse model, which expresses the human FTD Tau variant P301S, was found to exhibit elevated LD accumulation within microglia (187). Lysosomal dysfunction has been implicated in ALS/FTD pathology. LD accumulation in P301S tau mice ensues following the downregulation of neuronal adenosine monophosphate–activated kinase signaling, which regulates lipophagy by decreasing the expression of lipin-1 and glycerol-3-phosphate acyltransferase, key enzymes involved in lipogenesis (Fig. 3*H*) (187). Elevated peroxidated lipids are present in the sera of ALS patients, indicative of increased LDs (188). Analyses of lipids in the sera of ALS patients surprisingly revealed that elevated cholesterols and triglyceride levels correlate with increased lifespan (189). This discrepancy mirrors results observed in AD models, where maintenance of lipid homeostasis may be more protective than complete elimination of LDs. Together, these studies indicate that the pathogenic proteins involved in ALS and FTD affect lipid metabolism. Lipid dysregulation in turn alters homeostatic microglia functions including impaired phagocytosis of aggregated proteins, leading to a vicious cycle of neuronal damage and detrimental neuroinflammatory responses.

### LDs in MS

As described above, MS is characterized by focal demyelination lesions wrought by autoreactive T-cell responses, culminating in neurodegeneration and progressively worsening motor deficits. Lesion-resident microglia can clear myelin debris and secrete trophic factors to support remyelination (190). However, they can also perform disease-amplifying functions by secreting cytokines that can attract peripheral immune cells and amplify pro-inflammatory responses. Foamy, lipid-laden microglia have been observed near both acute and chronic MS lesions in MS postmortem brains (191, 192). A major source of lipids found inside these microglia are derived from myelin debris during demyelination. Accumulation of myelin debris prevents lipolysis which in turn triggers pro-inflammatory signaling. As with impaired aggregated protein phagocytosis, myelin containing microglia exhibits reduced capacity to phagocytose debris, compromising their protective/homeostatic functions. Reduced debris clearance consequently leads to the formation of cholesterol crystals that can activate the NLRP3 inflammasome (Fig. 3*I*) (193). Plin2-deficient mice, which exhibit diminished propensity for LD accumulation, were protected from neuroimmune responses when subjected to the cuprizone mouse model of demyelination (194). In postmortem MS tissue, macrophages containing myelin also expressed anti-inflammatory markers, CCL18, TGFβ, and IL-10 (Fig. 3*I*) (195). Further evidence for the protective role of LDs shows that alleviation of ER stress from LDs was required for remyelination (196).

Taken together, there is strong data to show that lipids influence many processes implicated in neurodegenerative disorders including protein misfolding, mitochondrial dysfunction, and modulation of neuroinflammatory responses. Acting as triggers for sterile inflammation, peroxidated lipids can engage and activate inflammatory pathways like the NLRP3 inflammasome and cell death pathways. However, LDs are not strictly harmful and may play a protective role in neurodegenerative disorders by alleviating ER and mitochondrial stress. Further research into therapeutics that can balance the protective functions of LDs with the deleterious effects of triggering neuroinflammation could serve as neuroprotective strategies across diverse neurodegenerative disorders.

## GWAS implicated hits constitute a linked innate immune pathway in AD

To date, over 75 loci have been implicated by GWAS studies as putative enhancers of AD risk (11). Genes identified as risk factors fall into a few categories dysregulated in AD including lipid metabolism, lysosomal dysfunction, and immune pathways. Several GWAS-implicated risk genes were found to exhibit microglial-specific/predominant expression signature (197). Remarkably, studies that have explored the biology of these proteins suggest that they may be part of a conserved pathway, which we will elaborate upon in this section.

### TREM2

**Figure 4:**
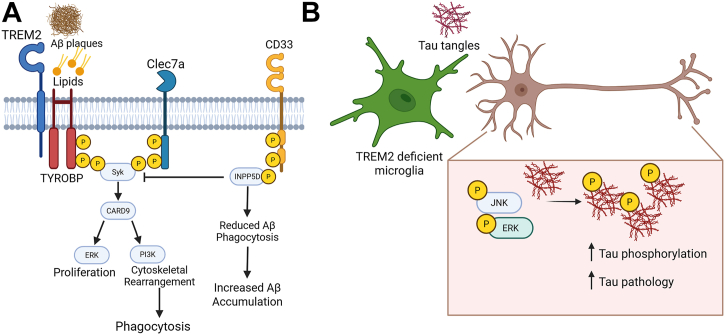
**GWAS-implicated targets reveal a conserved, microglia-resident innate immune mechanism that partakes in AD pathogenesis**. Identification of genetic risk factors for AD has uncovered several receptors that converge to form two opposing pathways modulating microglial responses to Aβ plaques. *A*, TREM2 and Clec7a recognize Aβ plaques and lipids leading to phosphorylation within their intracellular ITAM domains. This results in the autophosphorylation and activation of Syk kinase, resulting in Syk-mediated CARD9 phosphorylation. CARD9 interacts with ERK to increase cell proliferation and with PI3K resulting in cytoskeletal rearrangement and phagocytosis. CD33 is activated by sialylated proteins, including Aβ, leading to the phosphorylation of ITIM domains that phosphorylate INPP5D. INPP5D acts in opposition to Syk signaling by blocking its phosphorylation. Ultimately, INPP5D activation reduces microglial Aβ phagocytosis, leading to decreased Aβ plaque clearance. Together, the balance between these opposing pathways is disrupted in AD, increasing Aβ pathology and ensuing neuropathology. *B*, TREM2 exhibits disparate functions in the setting of Tau pathology. In humanized Tau overexpressing TREM2-deficient mice, increased expression of neuronal JNK and ERK results in increased tau phosphorylation and tau pathology. AD, Alzheimer's disease; Aβ, amyloid beta; CARD9, caspase-recruitment domain family member 9; GWAS, genome-wide association studies; INPP5D, inositol polyphosphate-5-phosphatase; ITAM, immunoreceptor tyrosine-based activation motif.

TREM2, a scavenger receptor exclusively expressed on microglia in the CNS, is an identified genetic risk factor for AD (13). TREM2 ligands include lipids such as phospholipids released by apoptotic cells and cholesterol esters, as well as protein aggregates (198, 199). Multiple TREM2 variants associated with increased AD risk limit ligand binding and receptor signaling. The R47H and R62H mutations reduce TREM2 ligand binding, increasing the risk of developing late onset AD by 2-3-fold (13, 200). Mechanistically, TREM2 signaling regulates multiple microglial functions including phagocytosis and cytokine secretion. TREM2 deficiency in mice results in increased Aβ accumulation as well as increased plaque-associated microglia when crossed with 5xFAD and APP/PS1 mice (201, 202, 203). Overexpression of TREM2 culminates in attenuated Aβ pathology and microgliosis, indicating that TREM2 is sufficient for microglial response to Aβ plaques (Fig. 4*A*) (204). TREM2 signaling was found to contribute to the induction of anti-inflammatory cytokines (205). TREM2-deficient, 5xFAD mice exhibit a dampened neuroinflammatory transcriptional profile, but this led to increased Aβ accumulation due to an attenuated ability of Trem2-deficient microglia to compact plaques (202). However, there is evidence for protective effects of loss of TREM2 signaling in AD models. TREM2-deficient, APP/PS21 transgenic mice showed reduced brain inflammation, which was found to rescue amyloid and Tau proteopathies (206). More research is needed to unravel the complexities of TREM2 signaling in the context of cytokine signaling, proteopathy, and neurodegeneration in AD models. Additionally, research into the role of TREM2 in Tau pathology highlights disparate and apparently conflicting roles as compared to Aβ pathology (Fig. 4*B*). Humanized Tau overexpressing, TREM2-deficient transgenic mice exhibit exacerbated tau aggregation and phosphorylation through neuronal upregulation of Tau kinases (207). In the P301L Tau model, TREM2 deletion does not show brain atrophy; however, in a double transgenic mouse model where progressive Tau aggregation is driven by mutant Aβ, Trem2 deficiency amplified Tau oligomerization and spreading, culminating in brain atrophy (Fig. 4*B*) (208). Consistent with these results, TREM2 deficiency or R47H variant expression was found to facilitate Tau spreading and proteopathy (209). APOE4-associated elevated Tau aggregation was exacerbated upon TREM2 deletion (210). To conclude, initial studies implicated TREM2 in Aβ binding/internalization and degradation (29). TREM2 was found to engage pro-inflammatory signaling cascades that led to context and model-dependent beneficial as well as detrimental roles in AD pathology.

### Clec7a

Clec7a, also known as Dectin-1, is a surface receptor upregulated in plaque-associated microglia and AD tissues (211). The first reported ligand for Clec7a was fungal beta-glucans. In the context of AD, Clec7a can bind to Aβ aggregates leading to the activation of immunoreceptor tyrosine-based activation motif domains in TREM2 (212). Activation of Clec7a results in subsequent Syk phosphorylation, ultimately leading to Aβ phagocytosis and plaque compaction (Fig. 4) (213). It is currently unknown whether/how Clec7a signaling affects Tau aggregation and neuropathology.

### Syk

Syk is a nonreceptor tyrosine kinase that plays a role in innate immune signaling pathways in the peripheral immune system as well as in resident immune cells of the CNS. Recent investigations indicate that Syk, a downstream kinase activated by TREM2 and Clec7a, functions within the conserved AD-associated signaling pathway. Microglial-specific Syk depletion in 5xFAD mice resulted in increased plaque-associated microglia and Aβ accumulation due to reduced microglial phagocytic activity (213, 214). In response to Aβ plaques, Syk can be sequestered within stress granules, reducing signal transduction from phagocytic receptors including TREM2 and increasing Aβ load (215). Phosphorylated Syk partakes in innate inflammatory signal cascades that regulate cytokine release and cell survival. Aβ oligomers were found to increase Syk phosphorylation leading to subsequent activation of the NLRP3 inflammasome and deactivation of the adenosine monophosphate–activated kinase pathway (216). Syk inhibition effectuated a reduction in amyloid plaque burden and tau tangles by blocking NLRP3 inflammasome activation (216). Together, these studies demonstrate that disruption of this TREM2–Syk signaling axis increases pathologic protein accumulation and AD risk.

### Caspase-recruitment domain family member 9

CARD9 is an adaptor molecule that plays a role in myeloid cell responses to fungal infection (217). CARD9 relays signals from surface receptor activation and activates the key innate immune transcription factor, NF-κB. Additionally, it can interact with other CARD-domain–containing proteins including ASC, caspase-1, among others, implicating CARD9 in inflammation, pyroptosis, and apoptosis. In the brain, CARD9 is almost exclusively expressed in microglia (218). Fungal infections in the CNS result in Clec7a- and Syk-induced CARD9 activation, culminating in increased IL-1β secretion (219). The convergence of GWAS-identified microglial receptors on Syk signaling suggests that CARD9 may play a role in AD pathology. Card9^−/−^/5xFAD mice had increased Aβ burden and increased microgliosis (218). While CARD9 deletion was found to regulate microglial proliferation and cytokine induction in these settings, it did not impact microglial ROS production or LD accumulation (218).

### CD33

CD33 is a transmembrane receptor expressed predominantly on microglia that recognizes sialic acid that is upregulated in AD tissue (220). Aβ plaques can aggregate with sialylated proteins, allowing for CD33 recognition of plaques, triggering a signaling cascade that inhibits microglial phagocytosis (221). Transgenic ablation of CD33 in 5xFAD mice reduced Aβ load (222). The rs3865444 CD33 variant reduces AD risk, through decreased CD33 expression and increased Aβ phagocytosis and degradation (220). Activation of CD33 results in phosphorylation of its immunoreceptor tyrosine-based inhibitory motif (ITIM) domains, recruiting inositol polyphosphate-5-phosphatase (INPP5D).

### Inositol polyphosphate-5-phosphatase

INPP5D is activated by phosphorylation of the ITIM domains on CD33 (223). Primarily expressed in microglia, INPP5D inhibits Syk signaling by sterically blocking the interaction between Syk and the adaptor protein, Tyrobp (Fig. 4*A*) (224). GWAS analyses have identified intronic SNPs which confer a higher risk of AD (225, 226). INPP5D levels were induced within microglia in AD tissue (227, 228) as well as in transgenic 5xFAD mice and PS19 transgenic tau mouse lines (224, 228, 229). Inhibition of INPP5D resulted in activation of the NLRP3 pathway through regulation of NFκB in human iPSC microglia (227). Additionally, transcriptomic profiling of INPP5D haploinsufficient mice showed upregulation in genes that regulate cell migration, phagocytosis, and wound healing (228, 230). Consistent with these results, transgenic INPP5D ablation results in increased plaque-associated microglia and plaque compaction in efforts to contain Aβ plaques (224, 231, 232). Together, these findings suggest that microglial INPP5D contributes to plaque formation in the settings of AD.

### Summary of GWAS identified conserved signaling pathways

Upon activation of TREM2, the adaptor protein DAP12 auto phosphorylates at its immunoreceptor tyrosine-based activation motif domains, leading to recruitment and subsequent phosphorylation of Syk. Phosphorylation of Syk promotes activation of the ERK pathway, resulting in increased cell proliferation, and PI3K/AKT activation. On the other hand, CD33 activation results in autophosphorylation of ITIM domains that recruit and phosphorylate INPP5D. Phosphorylated INPP5D inhibits Syk by blocking its interaction with the Syk adaptor protein, TYROBP, effectively shutting down TREM2 signaling (224). Together, these studies in other neurodegenerative disorders implicate TREM2 signaling in multiple aspects of pathogenesis.

## GWAS-implicated pathways in other neurodegenerative diseases

Thus, GWAS identification and subsequent studies have converged on two pathways that modulate multiple aspects of AD pathology. While initially identified and studied in the context of AD, emerging evidence has implicated some of these genes in disease progression of other neurodegenerative disorders, suggesting that they might contribute to multiprotein neuropathology load. For example, mutations in TREM2 have been associated with PD (233), FTD (13, 233), ALS (234), and MS (235). However, the molecular mechanisms of TREM2 mutations in pathophysiology of these diseases remain uncharacterized. Both the EAE model of MS and cuprizone demyelination model have shown TREM2 facilitates removal of myelin debris and increases remyelination (236, 237). In PD, TREM2 KO mice exhibited elevated microgliosis and exacerbated dopaminergic neuron loss when subjected to an AAV-αSyn overexpression (238). αSyn-PFF–injected TREM2-deficient mice exhibited exacerbated αSyn pathology when compared to their WT counterparts (239).

The studies covered in this section highlight how GWAS-implicated genetic loci can subsequently be linked to disease pathogenesis, which has largely been accomplished in the settings of AD biology. With similar large-cohort studies also emerging for other brain disorders such as PD, ALS, and FTD, it is conceivable that genomics could shine the light on novel disease-associated pathways that open new avenues of research/therapeutics for these disorders.

## Conclusion

The central nervous system is characterized by the systematic assembly of neuronal circuits. Long-lived neuronal cells are vulnerable to oxidative and proteopathic insults. Innate-immune signaling mechanisms have been shown to be intimately involved in both the maintenance of CNS architecture, as well as the collapse of neuronal circuitry in the settings of neurodegenerative disease.

Microglia and astrocytes serve critical roles in clearing myelin debris, potentially harmful metabolic byproducts including peroxidated lipids, as well as protein aggregates that can trigger neuronal cell death. On the other hand, neuroinflammatory pathways can become chronically active, secreting cytokines and other factors that perpetuate chronic inflammation, and amplify neuropathology in the settings of disease. In the peripheral immune system, quick cell turnover prevents chronic inflammation by replacing chronically active immune cells with quiescent cells. However, in the CNS, immune cell half-life is on the scale of years to decades (240). Because the CNS lacks this turnover mechanism, it is conceivable that unchecked/unabated neuroinflammatory signaling contributes to neuron death and dysfunction. In this review, we have summarized recent research on innate-immune signaling mechanisms that have been implicated in neurodegeneration. This includes innate immune assemblies such as inflammasome complexes and the cGAS/STING pathway, damaging lipid droplets, and GWAS-implicated gene products that function in a conserved, AD-associated signaling pathway. Going forward, continued research into these novel pathways as well as other understudied neuroinflammatory pathways could identify potential disease-modifying therapeutic targets.

## Conflicts of interest

The authors declare that they have no conflicts of interest with the contents of this article.
